# Comparison of methods for transcriptome imputation through application to two common complex diseases

**DOI:** 10.1038/s41431-018-0176-5

**Published:** 2018-07-05

**Authors:** James J. Fryett, Jamie Inshaw, Andrew P. Morris, Heather J. Cordell

**Affiliations:** 1 Institute of Genetic Medicine, Newcastle University, Newcastle upon Tyne, UK; 2 JDRF/Wellcome Diabetes and Inflammation Laboratory, Wellcome Centre for Human Genetics, Nuffield Department of Medicine, University of Oxford, Oxford, UK; 3 Department of Biostatistics, University of Liverpool, Liverpool, UK

**Keywords:** Gene expression, Genetic association study

## Abstract

Transcriptome imputation has become a popular method for integrating genotype data with publicly available expression data to investigate the potentially causal role of genes in complex traits. Here, we compare three approaches (PrediXcan, MetaXcan and FUSION) via application to genome-wide association study (GWAS) data for Crohn’s disease and type 1 diabetes from the Wellcome Trust Case Control Consortium. We investigate: (i) how the results of each approach compare with each other and with those of standard GWAS analysis; and (ii) how variants in the models used by the prediction tools compare with variants previously reported as eQTLs. We find that all approaches produce highly correlated results when applied to the same GWAS data, although for a subset of genes, mostly in the major histocompatibility complex, the approaches strongly disagree. We also observe that most associations detected by these methods occur near known GWAS risk loci. Application of these transcriptome imputation approaches to summary statistics from meta-analyses in Crohn’s disease and type 1 diabetes detects 53 significant expression—Crohn’s disease associations and 154 significant expression—type 1 diabetes associations, providing insight into biology underlying these diseases. We conclude that while current implementations of transcriptome imputation typically detect fewer associations than GWAS, they nonetheless provide an interesting way of interpreting association signals to identify potentially causal genes.

## Introduction

Genome-wide association studies (GWAS) have been successful at finding regions of the genome associated with a range of phenotypes—over 50,000 unique associations across more than 3200 traits are listed in the NHGRI-EBI GWAS catalogue [1] as of February 2018. However, the causal genes and biological mechanisms underlying these associations often remain unclear [2]. SNPs driving association signals in GWAS risk loci tend to fall within regulatory regions of genes, and are enriched for expression quantitative trait loci (eQTLs) [3], suggesting a key role of genetically regulated gene expression in complex human traits. Indeed, this has been confirmed through the use of methods examining the co-localisation of SNPs at GWAS loci and expression signals [4, 5]. However, these methods are unlikely to capture the full signal of genetically regulated gene expression as they often focus on single SNPs/eQTLs instead of all SNPs near to a gene, and they may fail to detect small effect sizes of expression on a trait.

A recent approach to detecting effects of gene expression on traits by integrating genotype and expression data, known as transcriptome imputation, has been developed and implemented in the software packages PrediXcan [6], MetaXcan (an extension of PrediXcan) [7] and FUSION [8]. These methods have been used to identify potential expression associations with a number of traits, including schizophrenia, type 2 diabetes and autoimmune vitiligo [9–12]. A typical transcriptome imputation analysis consists of two steps. First, previously gathered resources where genotype and expression measurements have been taken from the same individuals, such as the Genotype-Tissue Expression (GTEx) Consortium [13], are used to construct models that predict values for the genetically regulated portion of gene expression from genotype data. Second, these predictive models are applied to data where both genotype and phenotype measurements have been gathered (individual-level data or summary statistics from GWAS) to impute expression values and test the association of expression values with the phenotype of interest. This approach has some conceptual similarity with two-sample Mendelian randomisation [14] for integrating genotype and expression data. It also offers a more direct test of the effect of gene expression on a phenotype than that performed by co-localisation methods such as *coloc* [4]. Crucially, by using resources, such as GTEx, where expression data have been gathered for multiple tissues, tissue-specific expression prediction models for tissues relevant to the phenotype of interest can be used.

Implementations of transcriptome imputation approaches have a number of methodological differences, such as the way the predictive models are built, and whether individual-level genotype data (as with PrediXcan) or summary statistics from GWAS (as with MetaXcan and FUSION) are used to perform the imputation and evaluate association with phenotype. Here, we compare transcriptome imputation methods by applying each of them to GWAS data from the Wellcome Trust Case Control Consortium (WTCCC1) study [15], focusing on Crohn’s disease (CD) and type 1 diabetes (T1D), and then comparing the results obtained. We also investigate the gene expression prediction models used by the different methods to understand better the differences between them. By using prediction models constructed using gene expression measures from the GTEx Consortium, we also compare how these methods perform when predicting expression-trait associations across a range of tissues relevant to each phenotype. As these methods have similarities to GWAS, we consider how they perform in comparison to standard GWAS analysis of the WTCCC1 data. Finally, we apply MetaXcan to summary statistics from recent larger meta-analyses of CD [16] and T1D [17] to find novel predicted expression-trait associations and to improve understanding of the biological mechanisms underpinning these diseases.

## Materials and methods

### WTCCC1 case–control data sets

The initial case/control data sets from WTCCC1 that we analysed corresponded to the same data sets used by Gamazon et al. [6]. These data consisted of 1748 CD cases, 1963 T1D cases and 2938 shared controls [15]. SNPs and samples that failed the WTCCC1-automated quality-control (QC) procedures, SNPs with MAF <0.01 and SNPs with abnormal cluster plots were removed. The remaining SNPs and samples were taken forward for genome-wide imputation. We followed the same genotype imputation procedure as Gamazon et al. [6], using the Michigan Imputation server [18], with the 1000Genomes phase 1 v3 reference panel (all ethnicities) and ShapeIT phasing. For each disease, we imputed cases and controls together. Following imputation, we removed SNPs with imputation *R*^2^ < 0.8 and MAF <0.01.

### Transcriptomic imputation-based methods

All transcriptome imputation-based methods examined here attempt to find potential gene expression-trait associations by predicting values for the genetically regulated portion of gene expression from SNP data, and then regressing predicted expression on the phenotype of interest. A typical analysis using these methods is performed in two steps. The first is the training of the gene expression prediction models in data sets where both genotype and gene expression data are available for the same individuals. This step has been performed by the developers of each method, and the resulting prediction models have been made available online. These models can be downloaded and applied to genotype data or GWAS summary statistics to impute gene expression-trait associations. PrediXcan and MetaXcan prediction models are available at http://predictdb.hakyimlab.org/, and the FUSION prediction models are available at http://gusevlab.org/projects/fusion/. Key differences between the software packages are described in Supplementary Table S1.

### Application of transcriptomic imputation methods

We applied PrediXcan [6] to the imputed WTCCC1 CD and T1D SNP data. When attempting to recreate the results of Gamazon et al. [6], we followed their analytical pipeline by using the subset of SNPs present in HapMap2 and gene expression prediction models trained in depression genes and networks (DGN) whole blood.

For comparative analyses between PrediXcan, MetaXcan and FUSION, we used the full set of imputed SNPs passing QC. For the analysis of CD, we applied prediction models for the following GTEx tissues: whole blood, Epstein–Barr virus (EBV)-transformed lymphocytes and sigmoid colon. For the analysis of T1D, we applied prediction models for the following GTEx tissues: whole blood, EBV-transformed lymphocytes and pancreas. eQTLs for these tissues (or similar tissues) have previously been used to investigate the role of gene expression in these phenotypes [5, 19, 20]. As MetaXcan and FUSION use summary statistics instead of individual-level genotype data, we performed GWAS on the imputed CD and T1D data using logistic regression implemented through SNPTEST [21]. We did not adjust for covariates. Association *z* scores were then used as input for MetaXcan and FUSION, as recommended by the software developers.

### eQTL data and comparisons

eQTL data for GTEx tissues analysed here were downloaded from the GTEx portal on 7 February 2017. For comparisons with gene expression predictive models from transcriptome imputation methods, we used eQTL data from the GTEx release that each set of models was based on—release V6p for PrediXcan/MetaXcan predictive models and V6 for FUSION predictive models. For each model, we calculated a measure of agreement between the eQTL data and the gene expression prediction model, *A*_eqtl_, which we defined as the percentage of SNPs present in both eQTL data and the predictive model and for which the direction of effect was the same in both of these.

### Geuvadis data and comparisons

Genotype and expression data for 465 individuals were obtained from the Geuvadis Project [22]. Genotypes were measured as part of the 1000 Genomes Project, and expression measurements were gathered via RNA-seq, then underwent quantile and PEER factor normalisation. We removed SNPs with MAF <0.01 or with imputation *R*^2^ < 0.8. We also removed African samples as their expression measures were markedly different to those of European samples, leaving genotype and expression data for 373 individuals. PrediXcan and FUSION models were applied to the genotype data to predict expression values, and correlation between these predicted expression values and the measured expression value was examined.

### Application of MetaXcan to published summary statistics

MetaXcan was applied to summary statistics from a recently published CD meta-analysis [16]. We used summary statistics from the meta-analysis of seven CD GWAS conducted as part of this study, but did not apply MetaXcan to the larger set of summary statistics as this included studies based on the Immunochip. Prediction models trained in 1000 Genomes data for the following GTEx tissues were used: whole blood, EBV-transformed lymphocytes and sigmoid colon.

We also applied MetaXcan to summary statistics from a recently published T1D meta-analysis [17]. We applied MetaXcan using prediction models trained in 1000 Genomes data for the following GTEx tissues: whole blood, EBV-transformed lymphocytes and pancreas.

## Results

### Replication of original PrediXcan results

As a proof of principle, we first aimed to recreate the findings of Gamazon et al. [6], by applying prediction models trained in DGN whole blood to the HapMap subset of SNPs from our imputed WTCCC1 CD and T1D data. Results are displayed in Supplementary Figure S1 and listed in Supplementary Table S2. Of the eight genes significantly associated with CD in Gamazon et al. [6], seven were significant in our results (Bonferroni corrected, *p* < 5.61 × 10^−6^), while *BSN* just missed significance (*p* = 1.10 × 10^−5^). Results were also similar for T1D, with 24 of 29 significant results in Gamazon et al. [6] achieving significance here. We found a much stronger peak of association in the MHC locus on chromosome 6 than Gamazon et al. [6] (most significant *p* in Gamazon et al. [6] = 2.92 × 10^−13^; most significant *p* in our results = 9.85 × 10^−71^), consistent with many T1D GWAS [15, 23]. We believe this difference likely occurred due to updates to the DGN prediction models between the application by Gamazon et al. [6] and our application here.

### Comparison of transcriptomic imputation-based methods

To compare how the different transcriptome imputation methods perform, we next applied PrediXcan, MetaXcan and FUSION to the imputed WTCCC1 CD and T1D data. For each method we applied predictive models trained in whole blood data from the GTEx Consortium [13], using the complete set of 1000 Genomes phase 1 imputed SNPs from the WTCCC1 data.

**Fig. 1:**
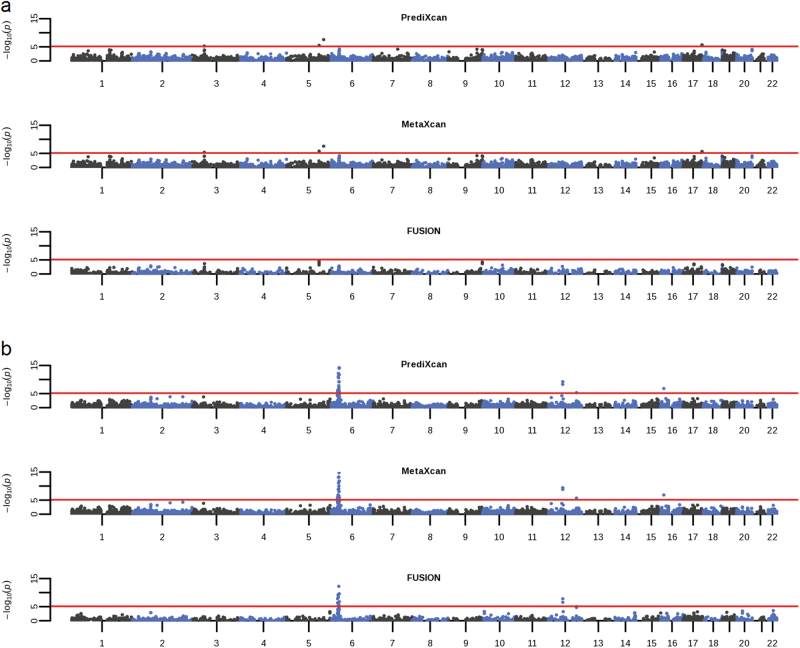
Comparison of results from applications of three transcriptome imputation methods to imputed WTCCC1 **a** CD data and **b** T1D data. Manhattan plots showing *p* values of predicted expression-trait associations from applications of PrediXcan, MetaXcan and FUSION to imputed WTCCC1 **a** CD data and **b** T1D data using prediction models trained in GTEx whole blood data. *P* values are plotted against the transcription start site for each gene. The red line on each plot shows the Bonferroni-corrected significance threshold at 7.44 × 10^−6^

The different approaches produced broadly similar sets of results when applied to the CD data (Fig. 1a). Both PrediXcan and MetaXcan found significant associations on chromosomes 3 (*C3orf62*), 5 (*SLC22A5* and *IRGM*) and 17 (*PGS1*), while FUSION found associations approaching significance on chromosomes 3 (*UBA7* in a similar position to *C3orf62*) and 5 (*SLC22A5*). *IRGM, PGS1* and *C3orf62* were not tested by FUSION, likely because each method used different criteria to determine the genes for which prediction models were built (Supplementary Table S1). Closer examination revealed that most differences between approaches’ results occurred because they tested different genes. For whole blood, PrediXcan and MetaXcan tested more genes (6105 genes) than FUSION did (2058 genes). In total, 1426 genes were tested by every method, representing a set of genes where direct comparison could be made.

Likewise, each method produced similar results when applied to the T1D data (Fig. 1b). All methods found significant associations at the MHC on chromosome 6, and on chromosome 12 (*RPS26* and *SUOX*). Again, many differences between the methods’ results could be explained by whether a gene was or was not tested. For example, predicted *CLEC16A* expression was found to be significantly associated with T1D by PrediXcan and MetaXcan, but was not tested by FUSION. Similarly, each method produced a strong peak of association at the MHC, but comprising of different genes—*CYP21A2* was found to be associated with T1D by PrediXcan (*p* = 3.26 × 10^−75^) and MetaXcan (*p* = 2.54 × 10^−93^), but was not tested by FUSION. In total, 23 MHC genes found to be significant by PrediXcan and MetaXcan were not tested by FUSION.

**Fig. 2:**
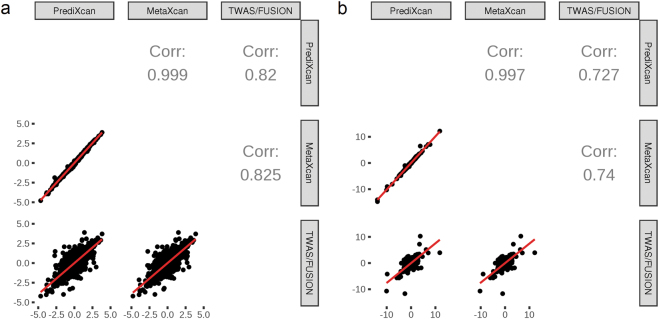
Pairwise correlations between results of transcriptome imputation methods for WTCCC1 CD data. Pairwise Pearson correlations between *z* scores produced by PrediXcan, MetaXcan and FUSION from applications to **a** imputed WTCCC1 CD data and **b** imputed WTCCC1 T1D data for the 1426 genes tested by all methods. Upper panels show correlation values, lower panels show scatter plots of *z* scores

We quantified the agreement between the different methods’ results, finding that *z* scores from the different methods were highly correlated (Fig. 2). As expected, PrediXcan and MetaXcan results showed the greatest correlation. Results from FUSION were also highly correlated with both PrediXcan and MetaXcan results, underlining the similarities between transcriptome imputation approaches.

For a number of genes, the methods produced notably different results, with PrediXcan/MetaXcan predicting different directions of effect of expression on phenotype compared to FUSION for 249 unique CD associations and 246 T1D associations. We determined the pairwise difference in *z* scores produced by each method for the 1426 genes analysed by every method, finding that all large differences in *z* scores between methods were between either PrediXcan and FUSION or MetaXcan and FUSION. Table 1 highlights genes showing large differences in *z* scores, where the methods disagreed the most about the effect of gene expression on the trait of interest. These differences are likely due to methodological differences between the different software packages (see Supplementary Table S1).

**Table 1:** Genes showing the three largest differences in MetaXcan *z* score and FUSION *z* score for each phenotype

Gene	Phenotype	PrediXcan *z* score	MetaXcan *z* score	FUSION *z* score
*GTF2H2C*	Crohn’s disease	−2.664	−2.74	1.483
*TAP2*	Crohn’s disease	3.011	2.961	−0.506
*PLD2*	Crohn’s disease	2.177	2.168	−0.87
*HLA-DQA1*	Type 1 diabetes	7.801	7.044	−17.338
*HLA-DQB1*	Type 1 diabetes	−2.961	−2.923	−21.825
*HLA-DRB1*	Type 1 diabetes	−7.735	−8.102	−20.594

To examine how well informed the gene expression imputation was for genes showing a large difference between their *z* scores achieved by the different software packages, we sorted the 1426 genes by the difference between their MetaXcan *z* score and FUSION *z* score. These genes were split into ten equally sized bins, with the 10% of genes with smallest differences in *z* scores in bin 1 and the 10% of genes with largest *z* score differences in bin 10. For each bin, the mean percentage of each PrediXcan/MetaXcan prediction model’s SNPs that were present in our post-QC GWAS data was calculated. PrediXcan/MetaXcan models in upper bins consistently had fewer of their models’ SNPs present in the GWAS data than PrediXcan/MetaXcan models for genes in lower bins (Supplementary Figures S2a and S2b). Interestingly, bin 10 showed a greater proportion of genes from the MHC on chromosome 6 than would be expected if bins were drawn randomly in both CD and T1D analyses (Supplementary Figures S3a and S3b).

To determine which method’s models best matched their expression training data, we next compared each model’s SNPs with eQTL statistics from the same GTEx data that were used to construct them. We hypothesised that predictive models would likely contain SNPs identified as eQTLs, and that these SNPs would show the same direction of effect on expression in the eQTL statistics as in the prediction models. This seemed likely as predictive models were based on a linear additive genetic model, so were unlikely to capture complex relationships such as SNP–SNP interactions. We tested each of the predictive models used by PrediXcan, MetaXcan and FUSION by calculating a measure of agreement between the model and eQTL data that we term *A*_eqtl_ (see 'Methods'). Supplementary Figure S4a shows the distribution of this statistic for each GTEx whole blood prediction model from PrediXcan/MetaXcan and FUSION. Prediction models for PrediXcan and MetaXcan always showed an *A*_eqtl_ of 1, indicating that every SNP had a direction of effect consistent with the eQTL data, and all but 14 FUSION models showed an A_eqtl_ of 1, showing near perfect concordance with eQTL data.

As a further comparison between methods, we applied PrediXcan and FUSION models to genotype data from the Geuvadis project to predict expression, and examined the correlation between predicted and measured expression levels. Overall, there were 952 genes for which both PrediXcan and FUSION predicted expression, and for which expression was also measured in Geuvadis. For these 952 genes, the prediction *R*^2^ values (i.e., the mean squared correlation coefficient between predicted and observed expression) obtained from PrediXcan and FUSION were found to be highly correlated (Supplementary Figure S5) although the actual values of *R*^2^ achieved were quite variable, with low values of *R*^2^ indicating limited predictive ability for many genes.

### Comparison of prediction models trained in different tissues

Each transcriptome imputation approach has made available predictive models derived from a range of GTEx tissues. To compare these, transcriptome imputation methods were applied to the imputed WTCCC1 data for CD and T1D using prediction models for GTEx tissues relevant to each phenotype—EBV-transformed lymphocytes and pancreas for T1D, and EBV-transformed lymphocytes and sigmoid colon for CD.

PrediXcan prediction models trained in different GTEx tissues produced similar sets of results when applied to CD data (Supplementary Figure S6a), although with less similarity than had been found between applications of different methods using the same tissue. Although few significant associations were observed, genes approached significance on chromosomes 3 and 6 in all tissues. As observed previously, many differences between results across tissues could be explained by whether or not genes were tested. For example, *SLC22A5* approached significance in both whole blood (*p* = 3.07 × 10^−6^, *β* = −0.41) and EBV-transformed lymphocytes (*p* = 3.85 × 10^−5^, *β* = −0.36), but was not tested in sigmoid colon. In total, 851 genes were tested in every tissue and *z* scores for these genes from each tissue showed mildly positive correlation (Supplementary Figure S6c). Likewise, MetaXcan and FUSION found similar results across different tissues (Supplementary Figures S7a, S7c, S8a and S8c).

Application of PrediXcan prediction models trained in different tissues to T1D data also produced sets of similar results (Supplementary Figure S6b). Significant associations were observed on chromosomes 6 (*C4A*, *SKIV2L*, *PSMB9* and *BTN3A2* in all tissues) and 12 (*RPS26* in all tissues). In total, 1013 genes were tested across all three tissues examined here. Pairwise correlation between *z* scores from each tissue showed positive correlation (Supplementary Figure S6d). Using prediction models for the same tissues, MetaXcan and FUSION also found results showing some similarities and with positive pairwise correlation (Supplementary Figures S7b, S7d, S8b and S8d). In each tissue, PrediXcan and MetaXcan consistently tested more genes than FUSION (Table 2), underlining the advantage PrediXcan and MetaXcan hold over FUSION as methods for discovering potential associations.

**Table 2:** Number of genes tested by each transcriptome imputation method

Tissue	Number of genes tested by PrediXcan/MetaXcan	Number of genes tested by FUSION	Number of genes tested by both methods
GTEx whole blood	6759	2058	1426
GTEx EBV-transformed lymphocytes	3759	1464	937
GTEx sigmoid colon	3859	1528	873
GTEx pancreas	4775	1691	1110

We repeated our comparisons of predictive models with results expected from eQTL statistics across all tissues tested here, finding that PrediXcan and MetaXcan consistently showed complete agreement with eQTL data (*A*_eqtl_ = 1 for all models), while almost all FUSION models showed complete agreement (Supplementary Figure S4). After repeating the process of binning genes according to the difference between their MetaXcan and FUSION *z* scores, higher bins consistently showed lower informativity for PrediXcan/MetaXcan models across all tissues (Supplementary Figure S2). Genes on chromosome 6 (specifically the MHC) were found in bin 10 more often than would be expected if bins were drawn randomly for all tissue–phenotype combinations except CD—EBV-transformed lymphocytes (Supplementary Figure S3).

### Comparison of detection ability of transcriptome imputation methods and GWAS

Transcriptome imputation methods are conceptually similar to GWAS and have been suggested as a complementary approach to GWAS. To investigate how these two approaches compare with respect to detection and localisation of associations, we performed standard GWAS on each of the imputed WTCCC1 CD and T1D data sets. Manhattan plots for these GWAS are shown in Supplementary Figure S9. Significant genes found by transcriptome imputation methods (most stringent Bonferroni threshold was *p* < 5.61 × 10^−6^ for PrediXcan with DGN prediction models) consistently co-localised with our observed GWAS hits (genome wide significance threshold *p* < 5 × 10^−8^). Nine of the 14 loci that attained genome-wide significance for either CD or T1D through GWAS showed no significant association signal for predicted expression. In contrast, only two loci significantly associated with predicted expression were not identified through GWAS, implying that transcriptome imputation may not be as powerful for the discovery of new associations as GWAS, and reinforcing its role as being complementary to (rather than a replacement for) GWAS.

### Application to CD meta-analysis

**Fig. 3:**
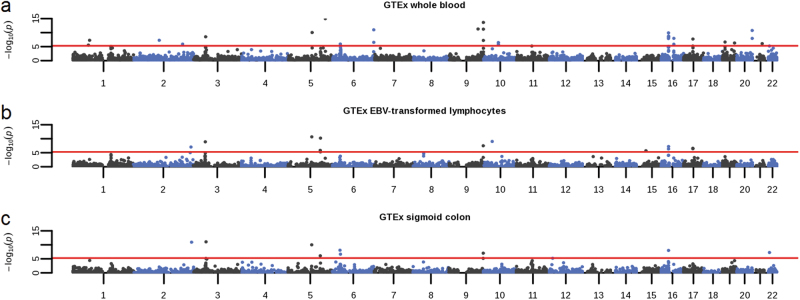
Application of MetaXcan to summary statistics from a meta-analysis of CD using prediction models for three tissues. Manhattan plots showing *p* values of predicted expression-trait associations from applications of MetaXcan to summary statistics from a CD meta-analysis using prediction models trained in **a** GTEx whole blood data, **b** GTEx EBV-transformed lymphocytes and **c** GTEx sigmoid colon. *P* values are plotted against the transcription start site for each gene. The red line on each plot shows the Bonferroni-corrected significance threshold at 5.15 × 10^−6^

We next applied transcriptomic imputation to a larger and better powered CD data set from a recent meta-analysis [16] comprising 5956 CD cases and 14,927 controls. As PrediXcan and MetaXcan showed slightly better agreement with eQTL data and tested more genes than FUSION, we chose to apply MetaXcan to these summary statistics. Gene expression prediction models for relevant GTEx tissues (whole blood, EBV-transformed lymphocytes and sigmoid colon) were used. In total, 54 unique predicted expression-trait associations passing genome-wide significance (*p* < 5.15 × 10^−6^) were identified in a range of genomic loci, with results displayed in Fig. 3 and significant associations listed in Supplementary Table S3. Of these 54 associations, 27 were predicted associations with gene overexpression and 27 with underexpression. On average, 92% of SNPs present in each predictive model were also in the meta-analysis, suggesting that predictions were accurate. Three of our detected associations had less than 50% of their prediction model’s SNPs present in the meta-analysis (*FLOT1*—whole blood, *FKBPL*—whole blood and *DDR1*—igmoid colon), so we urge caution when interpreting these results.

We observed 31 associations with predicted whole blood expression, 13 with EBV-transformed lymphocyte expression and 10 with sigmoid colon expression. Of our detected associations, 45 had been suggested previously in CD GWAS/meta-analysis [1], seven had not been suggested but were in GWAS risk loci, and two genes were in loci never before found to be associated with CD (*NPIPB6* and *NPIPB7*). The most significant association was with predicted *SLC22A5* expression in whole blood (*p* = 1.16 × 10^−16^, *β* = −0.40), and the association with the largest predicted effect size was *IFRD2* in sigmoid colon (*p* = 8.44 × 10^−12^, *β* = + 10.72).

### Application to T1D meta-analysis

**Fig. 4:**
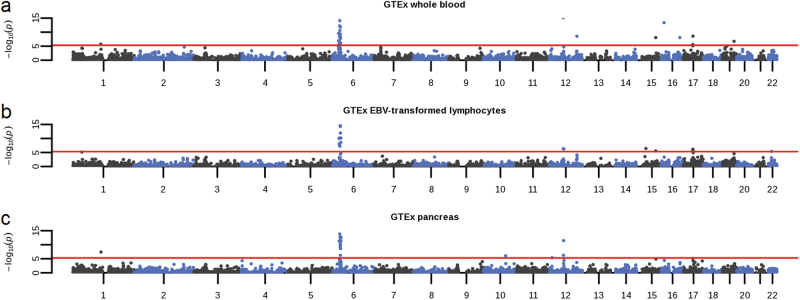
Application of MetaXcan to summary statistics from a meta-analysis of T1D using prediction models for three tissues. Manhattan plots showing *p* values of predicted expression-trait associations from applications of MetaXcan to summary statistics from a meta-analysis of T1D using prediction models trained in **a** GTEx whole blood data, **b** GTEx EBV-transformed lymphocytes and **c** GTEx pancreas. *P* values are plotted against the transcription start site for each gene. The red line on each plot shows the Bonferroni-corrected significance threshold at 5.01 × 10^−6^

MetaXcan was also applied to summary statistics from a recent meta-analysis of T1D [17] with 5913 cases and 8829 controls, using prediction models trained in GTEx whole blood, GTEx EBV-transformed lymphocytes and GTEx pancreas. A total of 154 predicted expression-trait associations reached genome-wide significance (*p* < 4.95 × 10^−6^), consisting of 69 overexpression associations and 85 underexpression associations. Most prediction models for significantly associated genes were well informed, with 74.1% of model SNPs present in the meta-analysis on average. Predictive models for ten significant associations (*C2*, *FLOT1*, *MICA*, *GABBR1*, *ZFP57* and *BAK1* in whole blood, and *ZFP57*, *ZSCAN31*, *PSORS1C1* and *MICA* in pancreas) had less than 50% of their SNPs in the meta-analysis, so we interpret these results with caution. Figure 4 shows Manhattan plots of these results, with significant associations listed in Supplementary Table S4.

Sixty-three significant associations were with predicted whole blood expression, 47 with predicted EBV-transformed lymphocyte expression and 44 with predicted pancreas expression, suggesting expression of genes across each of these tissues may be important in T1D. Significant associations tended to cluster in risk loci previously implicated in GWAS/meta-analyses of T1D, including the MHC (119 unique associations, including the 69 most significant associations) and 12q13.2 (7 unique associations), highlighting the importance of these regions in T1D. All but one (*ATP23*) of our detected associations had been previously suggested in a T1D GWAS/meta-analysis or were in a reported T1D risk locus [1]. The strongest association signal was with *HLA-DRB1* in pancreas, with *p* = 6.28 × 10^−305^. The association with the overall largest effect size (*p* = 8.04 × 10^−106^, *β* = − 34.36) was with *PBX2* expression in pancreas.

## Discussion

Transcriptome imputation has received much attention as a way of investigating the role of gene expression in complex disease. In this study, we have compared several transcriptome imputation approaches by applying them to previously-published GWAS of T1D and CD, and have shown that they produce similar results. Across the tissues and phenotypes examined here, we consistently observed that most predicted expression-trait associations overlapped with GWAS risk loci, showing the ability of this approach to identify potential causal genes within established GWAS risk loci.

Most differences between the methods’ results were due to different genes being tested. Each method used a different way of selecting which genes to produce models for. PrediXcan/MetaXcan models were created for all genes with expression values in GTEx, but only models where the correlation between predicted and observed expression was significant at an FDR <5% were uploaded, while FUSION models were created and uploaded for all genes whose SNP cis-heritability for gene expression was significant (*p* < 0.05). As genotype-expression data sets such as GTEx become larger, the number of genes for which prediction models can be built will almost certainly increase, improving the coverage of these methods.

We showed that for genes where the different methods disagreed most, a small number of SNPs in the prediction model used by PrediXcan and MetaXcan were missing from the GWAS data. For PrediXcan and MetaXcan, SNPs missing from the GWAS data set but in the predictive models are ignored during prediction. However, FUSION uses the ImpG-summary algorithm to impute summary statistics for these missing GWAS SNPs. While this may appear attractive, the GWAS SNPs were missing because they failed our post-imputation quality-control procedures, meaning FUSION’s imputation of these SNPs’ summary statistics likely has a high degree of uncertainty. For this reason, we believe PrediXcan and MetaXcan to be more compelling as their predictions are based only on the most reliable SNPs. Close attention should be paid to the proportion of a model’s SNPs that are present in the GWAS (or imputed GWAS) data for all methods.

By applying transcriptome imputation methods using prediction models for different tissues, we found that results were broadly similar across tissues, but with some crucial differences. Genetic regulation of gene expression is thought to be similar across GTEx tissues [13, 24], so it is reassuring to see our results recapitulate this. For this reason it is also likely that associations would be found even in tissues with little biological relevance to the phenotype of interest. We observed that many genes are tested across multiple tissues, raising the possibility of multivariate testing of gene expression incorporating tissue-tissue correlation. Multivariate testing is available (or being developed) in both the MetaXcan and the FUSION packages, and could improve power to detect genes whose expression is consistently associated with a phenotype across tissues.

We compared transcriptome imputation methods to the standard GWAS approach, finding that most significant associations occurred close to GWAS loci, implying that transcriptome imputation approaches are unlikely to detect many 'new' associations. However, by identifying potentially causal genes within each GWAS risk locus, transcriptome imputation still presents a useful complementary method to GWAS to aid downstream biological interpretation.

We found that PrediXcan, MetaXcan and FUSION performed similarly in terms of the prediction accuracy achieved. However, FUSION generally tests fewer genes, and FUSION’s imputation of summary statistics for missing SNPs (Supplementary Table 1) may result in predictions with low certainty. For these reasons, we chose to use MetaXcan for subsequent analyses.

We applied MetaXcan to summary statistics from a meta-analysis of CD, finding 54 associations between predicted gene expression and disease status. These included genes previously known to be associated with CD (*SLC22A5*, *IRGM* and *ATG16L1*), and genes that have never before been suggested (*NPIPB6* and *NPIPB7*). *NPIPB6* and *NPIPB7* are nuclear pore complex-interacting genes, however little else is known about them and further study is required to determine if a true causal role in CD is likely. Other interesting associations include *ETS2* and *ICAM1*, whose expression has been previously implicated in CD [25, 26]. A recent application of FUSION to the same CD summary statistics found similar results to those shown here [12], showing that similarities between transcriptome imputation approaches are not unique to the WTCCC1 data. Furthermore, SNPs near 34 of the 54 significant genes were found by 4C-seq to interact with DNA regulatory elements [27], underlining the potential role of genetically determined expression of these genes in CD.

Finally, we applied MetaXcan to summary statistics from a meta-analysis of T1D, finding 154 predicted expression-trait associations, including one (*ATP23*) mapping outside of known T1D risk loci. *ATP23* is thought to be involved in the repair of DNA double-strand breaks (DSBs), and further investigation of this finding is required to understand its potential role in T1D. Most of the other associations (including the 69 with strongest association signals) were with genes in the MHC. While associations in the MHC tend to be attributed to coding variation, it is also possible that this coding variation is in high linkage disequilibrium with regulatory variation that affects expression of genes involved in immunity. Due to the complex structure of linkage disequilibrium in the MHC, disentangling these possibilities is difficult, and more detailed investigation will be required. It is also possible that this complex structure of linkage disequilibrium and the large effect sizes typically seen in the MHC for T1D may have led to a number of spurious associations.

In conclusion, we have confirmed that transcriptome imputation is a powerful approach for interpreting the results of GWAS and identifying causal genes in common and complex diseases. We have shown that the current methods for transcriptomic imputation perform similarly, although PrediXcan and MetaXcan appear to be the most comprehensive. These approaches will be crucial for helping in the interpretation of GWAS results, and thus in the successful identification of future therapies for a range of complex diseases. It is likely that transcriptome imputation will become even more useful as improvements are made to each method and to genotype-expression reference panels.

## Electronic supplementary material

Supplementary Figure 1

Supplementary Figure 2

Supplementary Figure 3

Supplementary Figure 4

Supplementary Figure 5

Supplementary Figure 6

Supplementary Figure 7

Supplementary Figure 8

Supplementary Figure 9

Supplementary Table 1

Supplementary Table 2

Supplementary Table 3

Supplementary Table 4

Legends to supplementary figures and tables
