# Correction: Comparison of methods for transcriptome imputation through application to two common complex diseases

**DOI:** 10.1038/s41431-020-0605-0

**Published:** 2020-03-19

**Authors:** James J. Fryett, Jamie Inshaw, Andrew P. Morris, Heather J. Cordell

**Affiliations:** 1 Institute of Genetic Medicine, Newcastle University, Newcastle upon Tyne, UK; 2 JDRF/Wellcome Diabetes and Inflammation Laboratory, Wellcome Centre for Human Genetics, Nuffield Department of Medicine, University of Oxford, Oxford, UK; 3 Department of Biostatistics, University of Liverpool, Liverpool, UK

**Keywords:** Gene expression, Genetic association study

**Correction to:European Journal of Human Genetics**

10.1038/s41431-018-0176-5

The authors submitted the below correction to their original article. The original article has also been updated to reflect these changes:

Several bugs were recently discovered in some of the code used to run some of the analyses described in this paper. We deeply apologise for these errors.

The first analysis affected was the comparison of prediction models with eQTL data shown in Supplementary Fig. 4. A bug in the code meant that alleles were not being correctly matched up between the eQTL data and the FUSION models. This has now been fixed, and a corrected version of Supplementary Fig. 4 is shown below.

**Figure 1:**
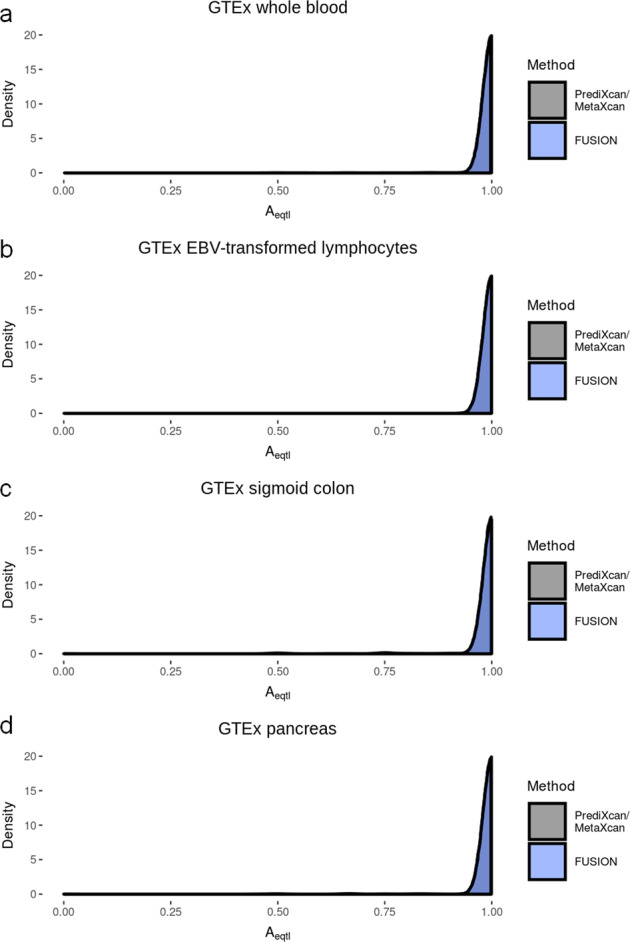
Supplementary Fig. 4

In this repeated analysis, we see that almost all FUSION models now show complete agreement with the eQTL data. We note that there are still a small number of models that do not show complete agreement, but as the difference between PrediXcan and FUSION models in this analysis is so small, we feel that our original conclusion changes. And so we must retract our statements made that “PrediXcan and MetaXcan’s models for predicting expression more consistently recapitulate known effects of genotype on expression” and “PrediXcan/MetaXcan models repeatedly showed better agreement with eQTLs than TWAS/FUSION” that were reported in the “Abstract” and “Discussion”.

The second analysis affected was the application of PrediXcan and FUSION prediction models to Geuvadis data shown in Supplementary Fig. 5. This analysis has now been fixed, and a corrected version of Supplementary Fig. 5 is shown below.

**Figure 2:**
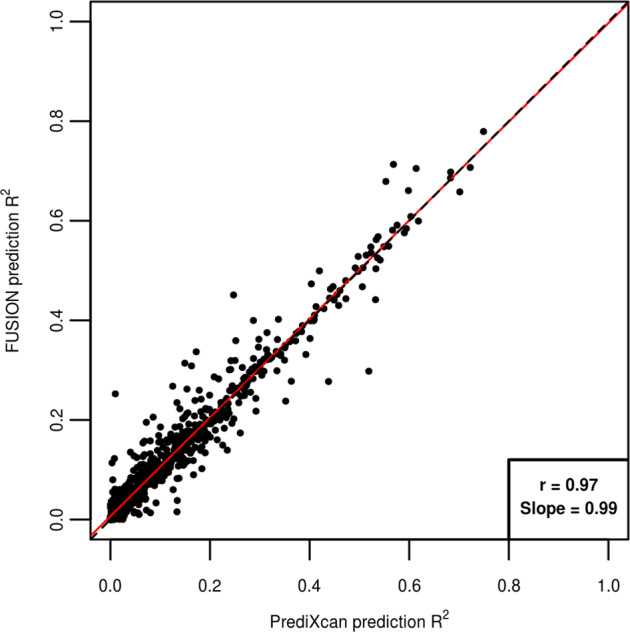
Supplementary Fig. 5

In the original manuscript, we reported that 643 genes were tested by both PrediXcan and FUSION when applied to Geuvadis data, and that the PrediXcan models achieved a prediction accuracy *R*^2^ of 0.061, while FUSION models achieved a prediction accuracy *R*^2^ of 0.05. In this corrected version, we now find that 952 genes were tested by both PrediXcan and FUSION when applied to Geuvadis data, with negligible difference seen between the prediction accuracies achieved. We therefore feel we must retract the statements made in the Abstract and manuscript respectively that “PrediXcan and MetaXcan generally produce more reliable results than FUSION”, “PrediXcan/MetaXcan models predict expression slightly more accurately”, and “PrediXcan and MetaXcan were consistently seen to be more robust than FUSION”.

The third analysis affected was the application of MetaXcan to summary statistics from a recent meta-analysis of Type 1 diabetes. The β coefficients (effect sizes) and *Z*-scores reported were accidently flipped from positive to negative (and vice versa) in Supplementary Table 4, meaning that our statement made in the manuscript of “85 overexpression associations and 69 underexpression associations” should actually read “69 overexpression associations and 85 underexpression associations”, and the association with the largest effect size has *β* = −34.36, not *β* = 34.36 as originally stated. A corrected version of Supplementary Table 4 has now been made available.

We apologise for these errors in our original manuscript.

