# Supplementary figures and images for: Comparison of methods for transcriptome imputation through application to two common complex diseases
Source: Eur J Hum Genet. 2018 Jul 5;26(11):1658–67. doi: 10.1038/s41431-018-0176-5 (PMC6189136; doi:10.1038/s41431-018-0176-5)

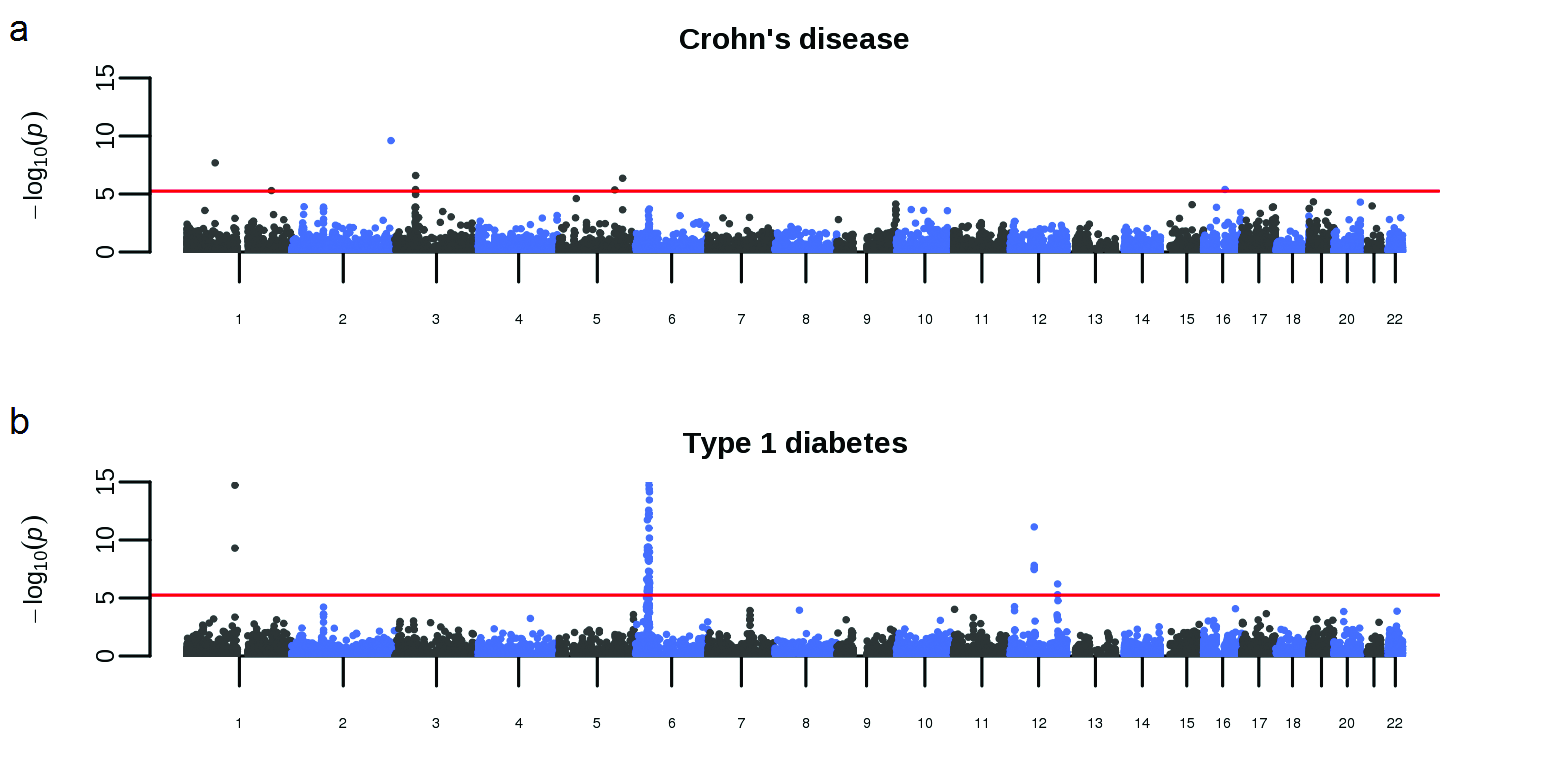

Supplement: Supplementary file 1 — Supplementary Figure 1 [file 41431_2018_176_MOESM1_ESM.tif]

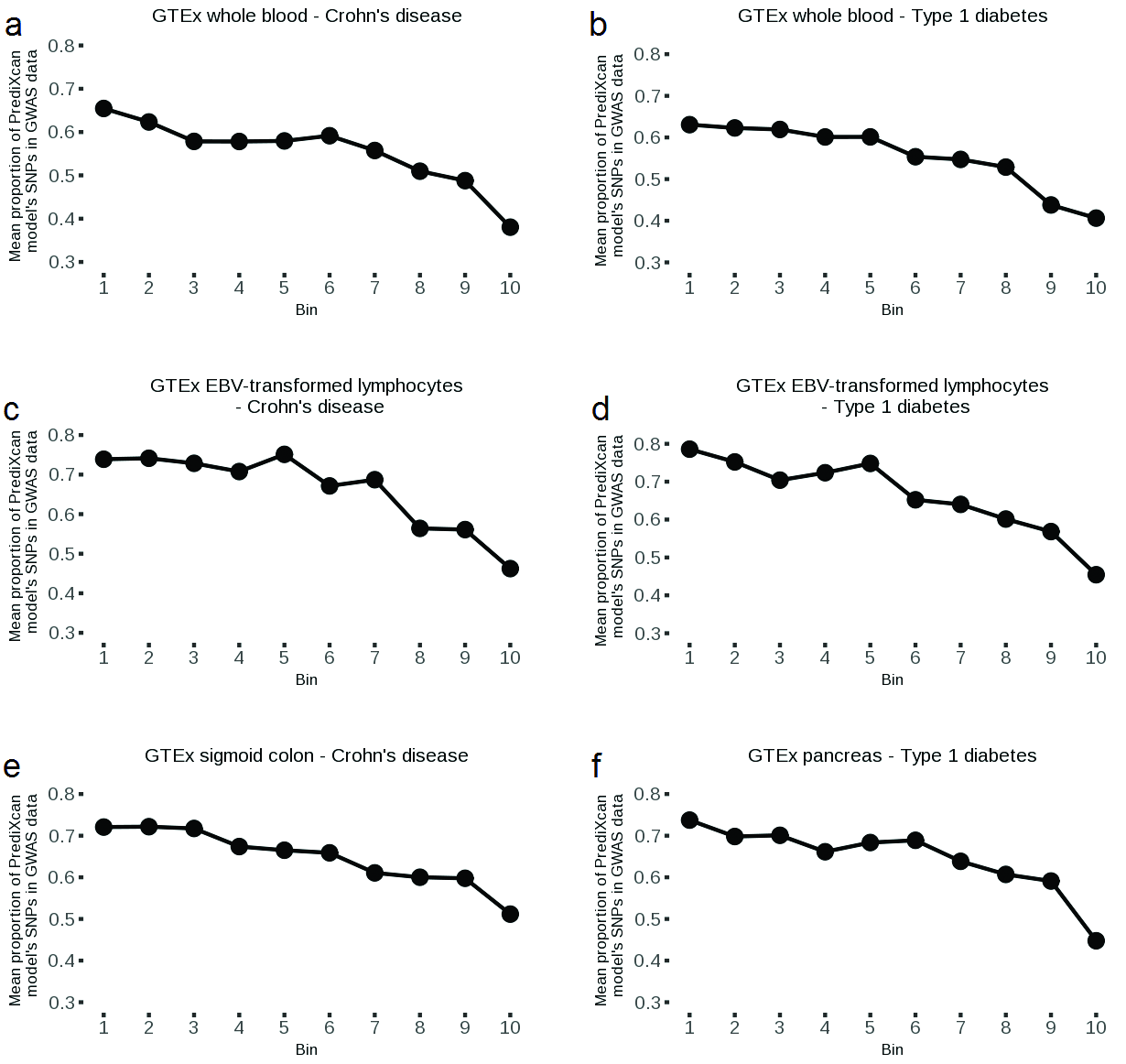

Supplement: Supplementary file 2 — Supplementary Figure 2 [file 41431_2018_176_MOESM2_ESM.tif]

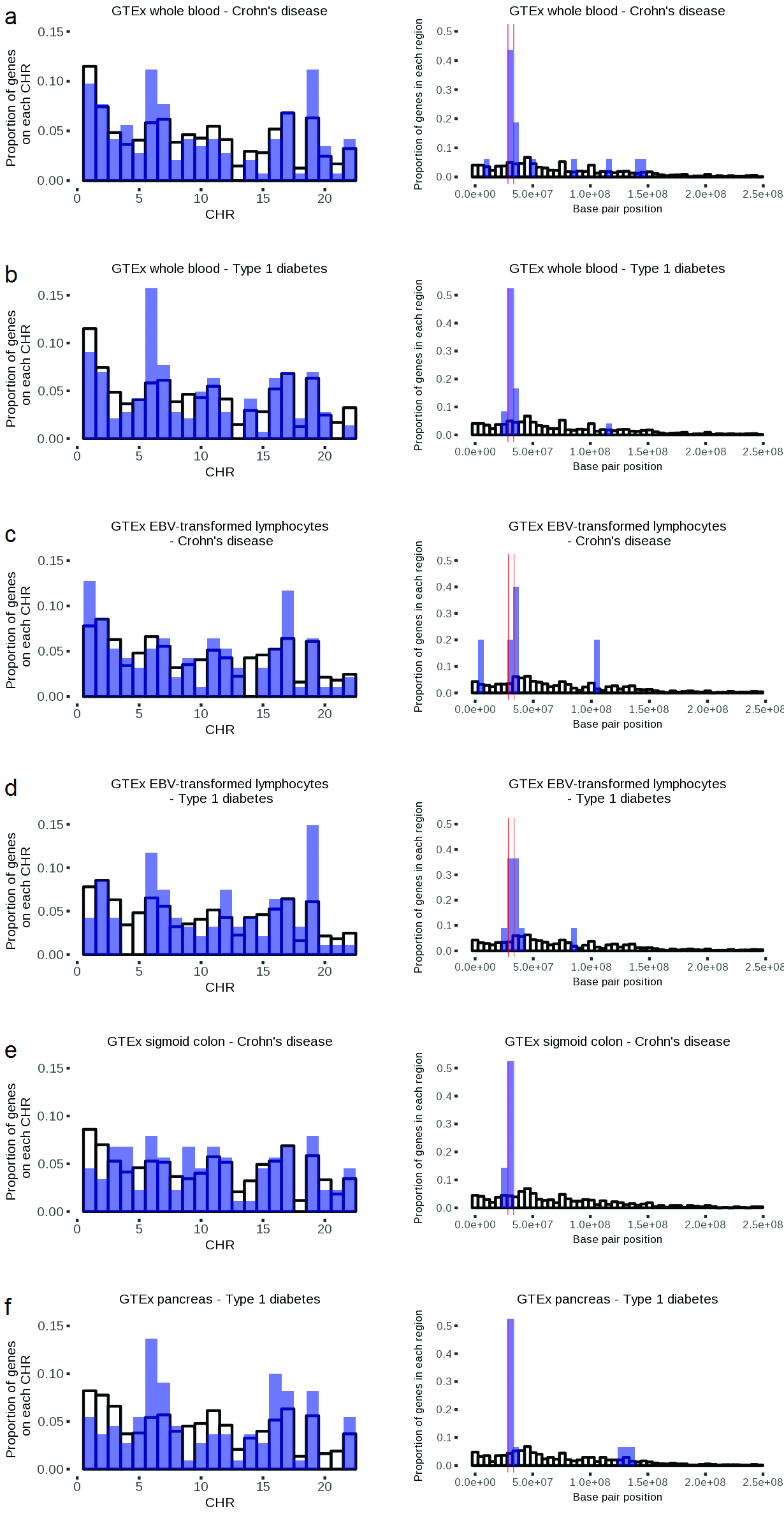

Supplement: Supplementary file 3 — Supplementary Figure 3 [file 41431_2018_176_MOESM3_ESM.tif]

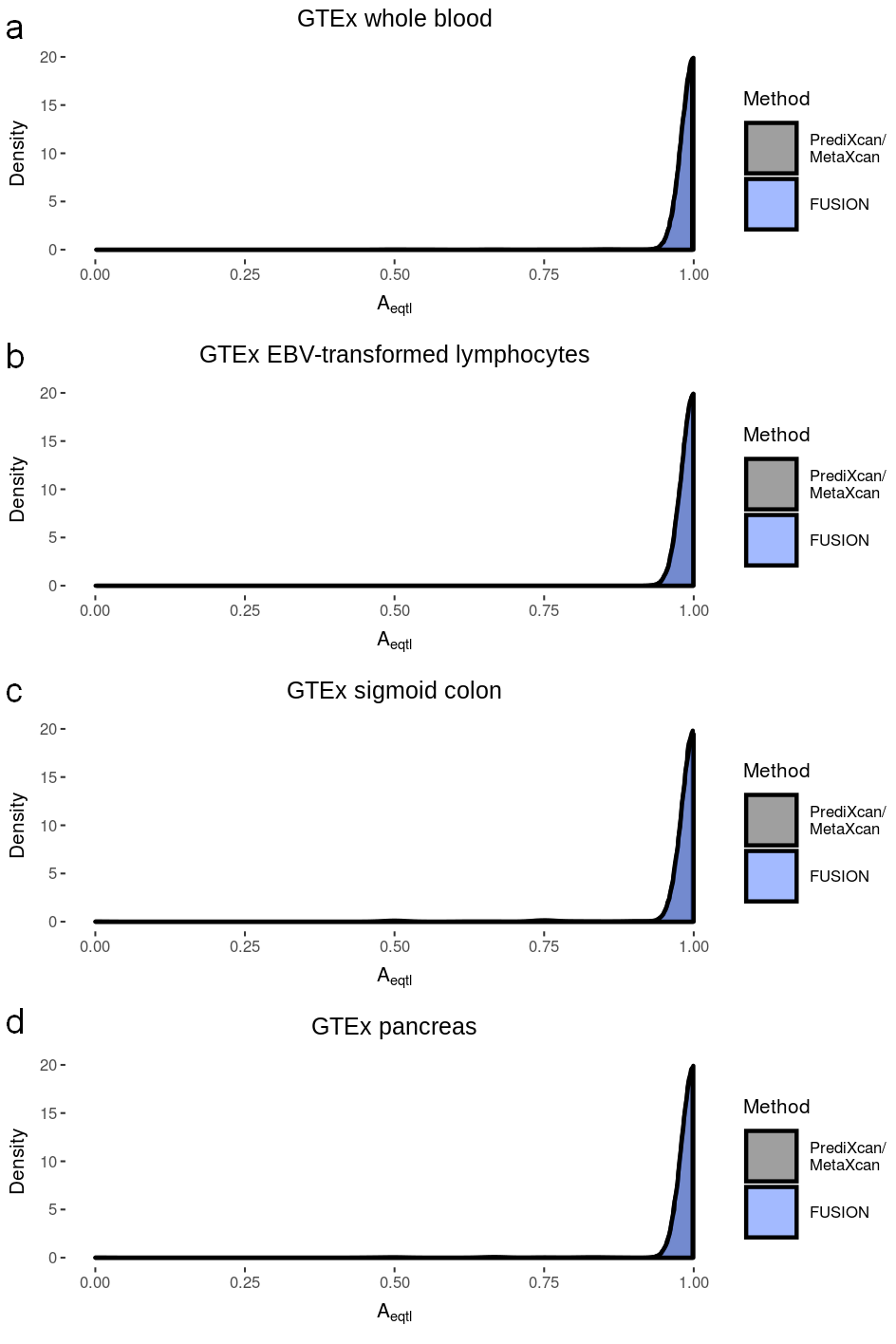

Supplement: Supplementary file 4 — Supplementary Figure 4 [file 41431_2018_176_MOESM4_ESM.tif]

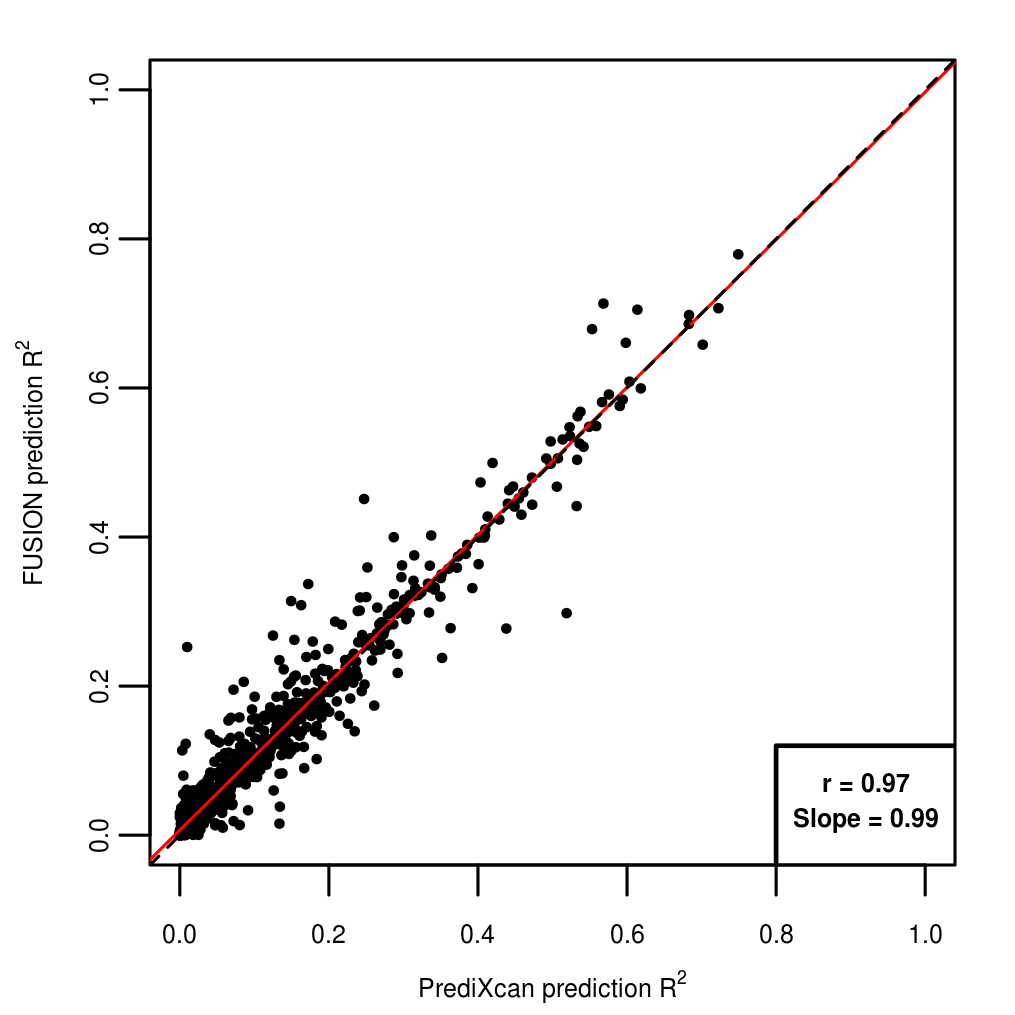

Supplement: Supplementary file 5 — Supplementary Figure 5 [file 41431_2018_176_MOESM5_ESM.tif]

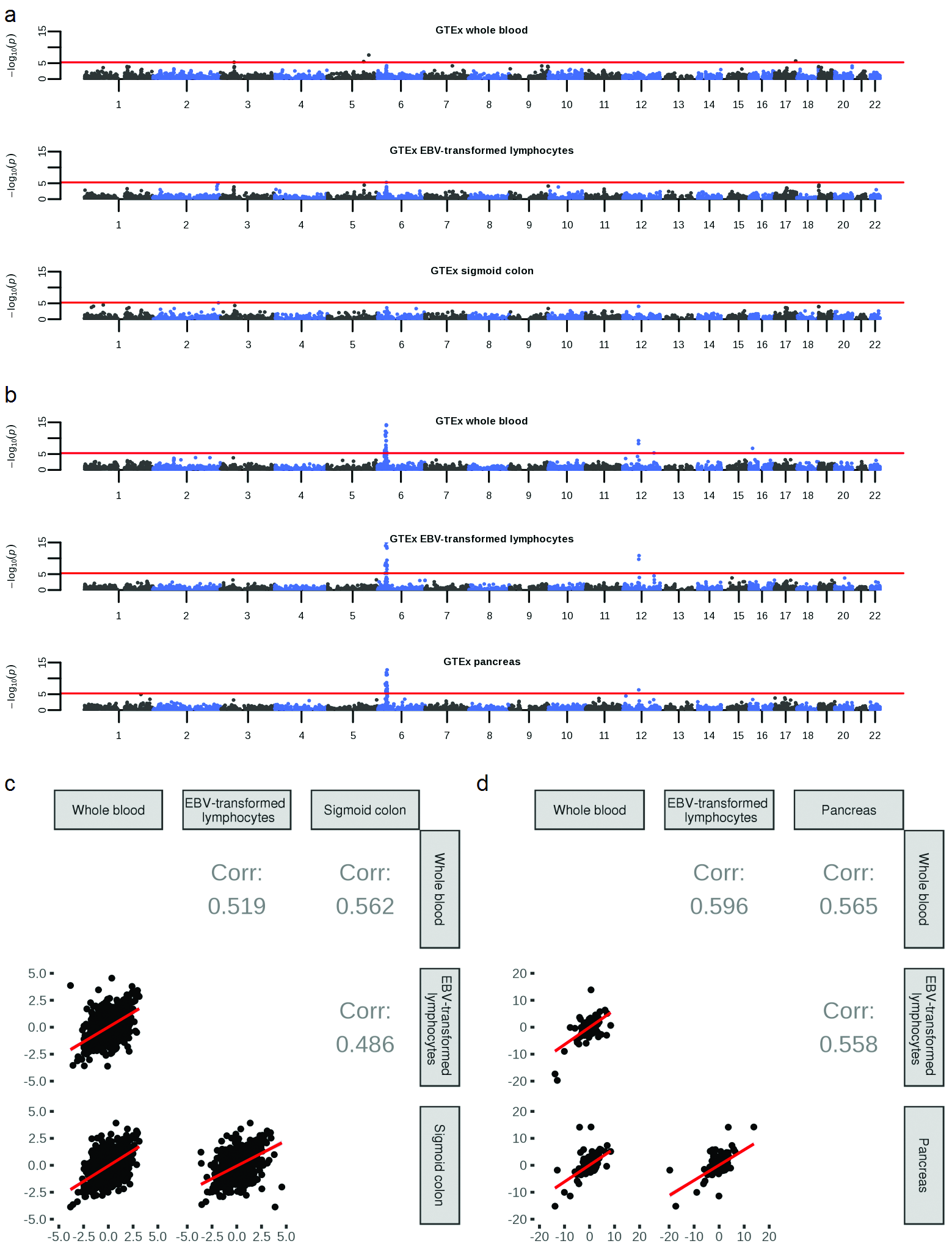

Supplement: Supplementary file 6 — Supplementary Figure 6 [file 41431_2018_176_MOESM6_ESM.tif]

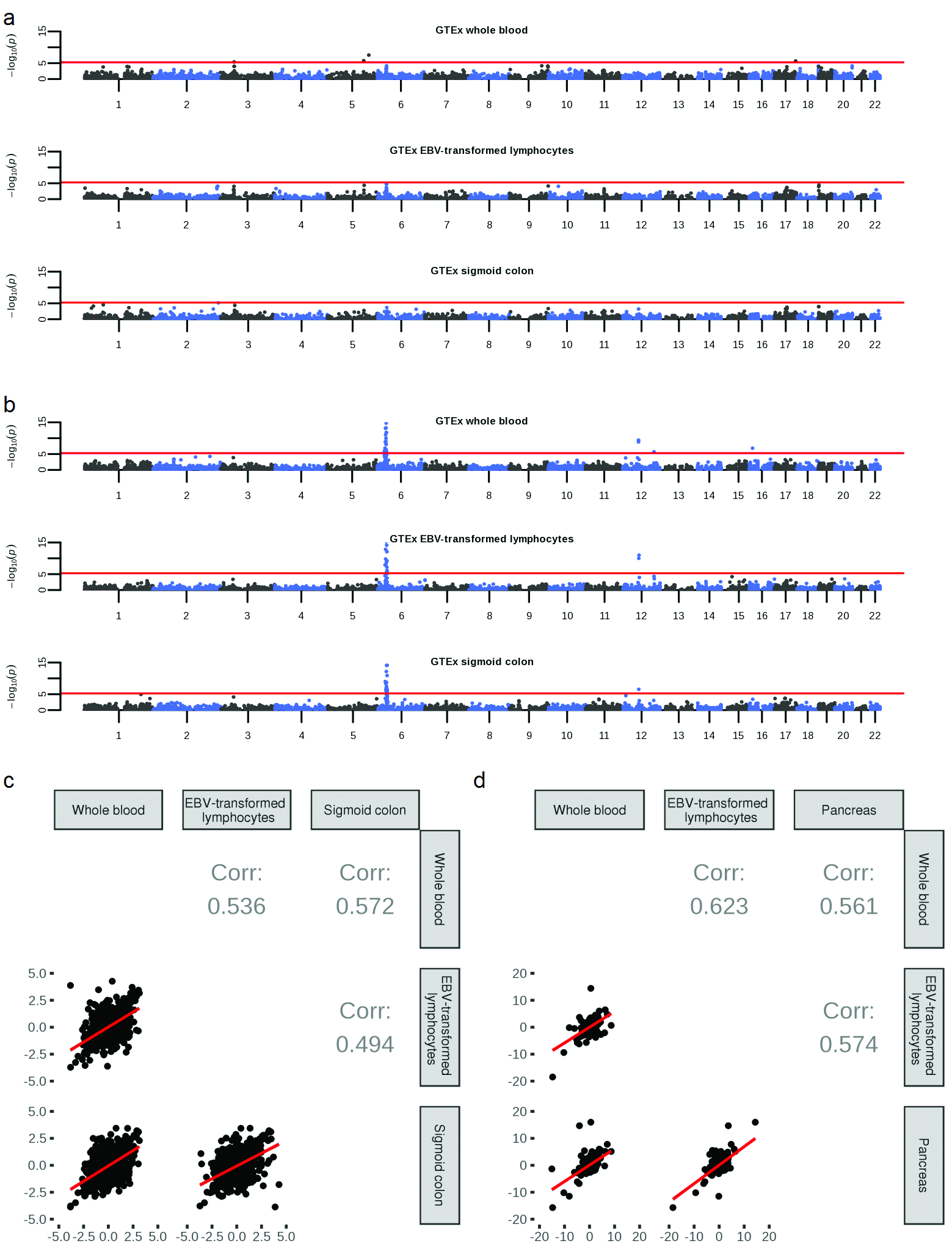

Supplement: Supplementary file 7 — Supplementary Figure 7 [file 41431_2018_176_MOESM7_ESM.tif]

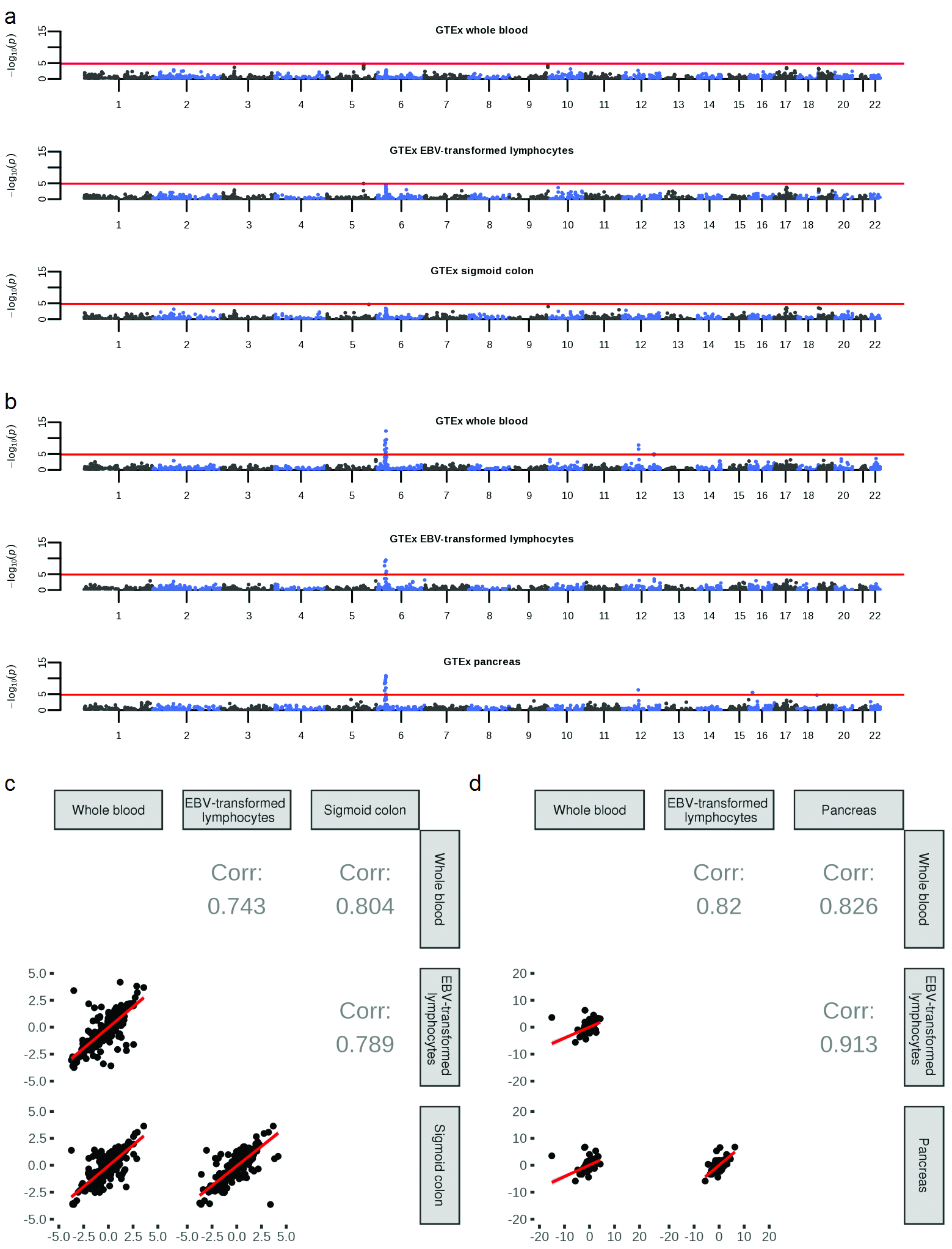

Supplement: Supplementary file 8 — Supplementary Figure 8 [file 41431_2018_176_MOESM8_ESM.tif]

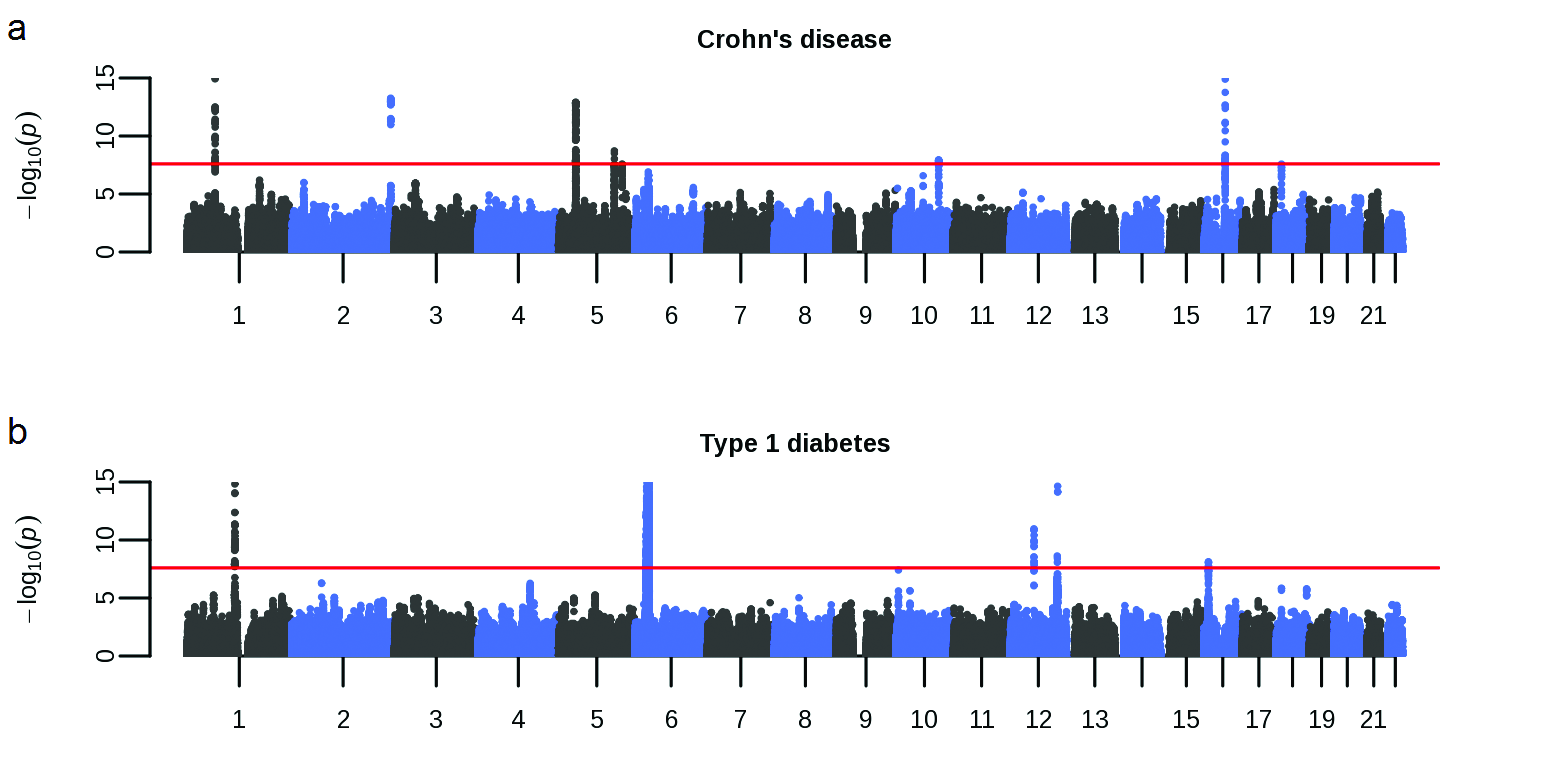

Supplement: Supplementary file 9 — Supplementary Figure 9 [file 41431_2018_176_MOESM9_ESM.tif]
