# Supplementary material for: Comparison of methods for transcriptome imputation through application to two common complex diseases
Source: Eur J Hum Genet. 2018 Jul 5;26(11):1658–67. doi: 10.1038/s41431-018-0176-5 (PMC6189136; doi:10.1038/s41431-018-0176-5)
Supplement: Supplementary file 14 — Legends to supplementary figures and tables [file 41431_2018_176_MOESM14_ESM.docx]

**Legends to supplementary figures and tables**

**Supplementary Figure 1. Recreation of original PrediXcan results.** Manhattan plots showing p-values of predicted expression-trait associations from applications of PrediXcan to (a) imputed WTCCC1 CD data and (b) imputed WTCCC1 T1D data using prediction models trained using DGN whole blood data, with the Bonferroni-corrected significance threshold at 5.61 x 10^-6^ for each phenotype. The y-axis has been cut off at p=1x10^-15^in each plot.

**Supplementary Figure 2. Informativity (**percentage of each model’s SNPs that were present in the imputed WTCCC dataset) **scores for PrediXcan/MetaXcan tests for genes tested by all methods, separated by tissue and phenotype, binned by the difference between z scores of different methods.**

**Supplementary Figure 3. Genomic distribution of genes in bin 10 for each tissue-phenotype combination.** Black-outlined bars represent the distribution of genomic locations of all genes tested for the specified tissue-phenotype combination, and blue bars represent the locations of genes in bin 10. Red lines show the boundaries of the MHC region (Ensembl GRCh37). Plots on the left show the distribution of genes in bin 10 across chromosomes, while plots on the right show the distribution of positions of genes on chromosome 6 in bin 10 along the chromosome, for each tissue-phenotype combination tested. Black-outlined bars represent the distribution of genomic locations of all genes tested for the specified tissue-phenotype combination. Blue shaded bars represent the distribution of genomic locations of genes in bin 10 for the specified tissue-phenotype combination. Red lines on plots on the right show the boundaries of the MHC region taken from Ensembl GRCh37.

**Supplementary Figure 4.** **Distribution of A_eqtl_ scores for PrediXcan/MetaXcan predictive models and FUSION predictive models for GTEx tissues.** A_eqtl_ scores were calculated for each PrediXcan/MetaXcan prediction model and FUSION prediction model, and the distribution of these scores was plotted for (a) whole blood, (b) EBV-transformed lymphocytes, (c) sigmoid colon and (d) pancreas.

**Supplementary Figure 5. Comparison of PrediXcan and FUSION from applications to Geuvadis data.** In the plot, each point represents one of the 643 genes for which PrediXcan and FUSION both predicted expression, and we had measured expression in Geuvadis. The x and y values for each point represents the squared correlation coefficient between measured expression, and expression predicted with either PrediXcan or FUSION. The dotted line is the line of equality, and the solid red line is the line of best fit. The correlation between x and y values and the slope of the best fit line are shown in the bottom right.

**Supplementary Figure 6.** **Comparison of results from applications of PrediXcan using prediction models based on 3 tissues to imputed WTCCC1 (a) CD and (b) T1D data, with pairwise correlations of PrediXcan z scores across tissues from this analysis shown for (c) CD and (d) T1D.** Manhattan plots showing p-values of predicted expression-trait associations from applications of PrediXcan to imputed WTCCC1 (a) CD data and (b) T1D data using prediction models trained in GTEx data for different tissues. P-values are plotted against the transcription start site for each gene. The red line on each plot shows the Bonferroni-corrected significance threshold at (a) 5.78 x 10^-6^ or (b) 5.51 x 10^-6^. Pairwise correlations between z scores from applications of PrediXcan to (c) CD data and (d) T1D data using prediction models trained in GTEx data for different tissues are also shown.

**Supplementary Figure 7.** **Comparison of results from applications of MetaXcan using prediction models based on 3 tissues to imputed WTCCC1 (a) CD and (b) T1D data, with pairwise correlations of MetaXcan z scores across tissues from this analysis shown for (c) CD and (d) T1D.** Manhattan plots showing p-values of predicted expression-trait associations from applications of MetaXcan to imputed WTCCC1 (a) CD data and (b) T1D data using prediction models trained in GTEx data for different tissues. P-values are plotted against the transcription start site for each gene. The red line on each plot shows the Bonferroni-corrected significance threshold at (a) 5.78 x 10^-6^ or (b) 5.51 x 10^-6^. Pairwise correlations between z scores from applications of MetaXcan to (c) CD data and (d) T1D data using prediction models trained in GTEx data for different tissues are also shown.

**Supplementary Figure 8.** **Comparison of results from applications of FUSION using prediction models based on 3 tissues to imputed WTCCC1 (a) CD and (b) T1D data, with pairwise correlations of FUSION z scores across tissues from this analysis shown for (c) CD and (d) T1D.** Manhattan plots showing p-values of predicted expression-trait associations from applications of FUSION to imputed WTCCC1 (a) CD data and (b) T1D data using prediction models trained in GTEx data for different tissues. P-values are plotted against the transcription start site for each gene. The red line on each plot shows the Bonferroni-corrected significance threshold at (a) 5.78 x 10^-6^ or (b) 5.51 x 10^-6^. Pairwise correlations between z scores from applications of FUSION to (c) CD data and (d) T1D data using prediction models trained in GTEx data for different tissues are also shown.

**Supplementary Figure 9.** **Manhattan plots of GWAS of imputed (a) WTCCC CD data and (b) WTCCC T1D data, with** the Bonferroni-corrected significance threshold at (a) 9.12 x 10^-9^ for CD and (b) 9.11 x 10^-9^ for T1D.

**Supplementary Table 1. Comparison of transcriptome imputation software packages.**

**Supplementary Table 2.** **P-values for genes significantly associated with CD or T1D from Gamazon et al., and their p-values in this analysis.**

**Supplementary Table 3.** **Predicted significant gene expression – Crohn’s disease associations from application of MetaXcan to meta-analysis summary statistics.**

**Supplementary Table 4. Predicted significant gene expression – type 1 diabetes associations from application of MetaXcan to meta-analysis summary statistics.**
